# MicroRNA-124-targeted recombinant Zika virus: a dual-functional and safe candidate for vaccination and oncolytic virotherapy

**DOI:** 10.1128/jvi.00208-26

**Published:** 2026-05-15

**Authors:** Chao Zhou, Meng-Li Cheng, Meng-Jiao He, Yao Liu, Yu-Yan Li, Dong-Yang Xie, Li-Shu Chen, De-Yu Li, Yong-Qiang Deng, Yan-Peng Xu, Qing Ye, Hui Zhao, Xiao-Feng Li, Qi Chen, Cheng-Feng Qin

**Affiliations:** 1 State Key Laboratory of Pathogen and Biosecurity, Academy of Military Medical Sciences 71040, Beijing, China; 2 Experimental Platform Management Office, Beijing Key Laboratory of Drug-Resistant Tuberculosis, Beijing Chest Hospital, Capital Medical University, Beijing Tuberculosis and Thoracic Tumor Institute 12517, Beijing, China; 3 State Key Laboratory of Proteomics, National Center of Biomedical Analysis, Beijing, China; University of North Carolina at Chapel Hill, Chapel Hill, North Carolina, USA

**Keywords:** Zika virus, microRNA-124, oncolytic virus, glioma, live attenuated vaccine

## Abstract

Zika virus (ZIKV) remains a significant public health threat due to its pronounced neurotropism linked to Congenital Zika Syndrome (CZS). Paradoxically, the same neurotropism also provides a unique opportunity for exploiting ZIKV as an oncolytic agent against malignant brain tumors such as glioma. However, achieving precise control of ZIKV replication in specific cell types or tissues remains a major challenge. Here, we show that the brain-specific and enriched microRNA, miR-124, is highly expressed in neural progenitor cells (NPCs) and neurons, but minimally expressed in glioma stem cells (GSCs) and differentiated glioma stem cells (DGCs). Based on these observations, we engineered a recombinant ZIKV containing a miR-124 target sequence (miR-124T) inserted into the 3′ untranslated region (UTR) of the viral genome, generating the virus designated ZIKV-miR124T. ZIKV-miR124T exhibited a significantly attenuated phenotype across multiple mouse models, including adult A129 mice, BALB/c neonates, and pregnant mice. Importantly, in an orthotopic glioma model, ZIKV-miR124T retained potent oncolytic activity while showing a markedly improved safety profile. Viral replication was strictly confined in the tumor region, with a ~1,000-fold reduction in viral load in non-tumoral brain regions compared to the well-established live-attenuated ZIKV (ZIKV-LAV). Furthermore, a single immunization with ZIKV-miR124T conferred effective protection against lethal ZIKV challenge and significantly reduced vertical transmission in pregnant mice. Collectively, our findings establish a strong proof of concept for a rational, miRNA-guided strategy to generate a next-generation ZIKV platform with dual potential as a safe live-attenuated vaccine and a precisely regulated oncolytic virus.

**IMPORTANCE:**

This study presents a crucial advancement in controlling the safety and function of neurotropic viruses. We engineered a dual-purpose ZIKV, ZIKV-miR124T, which is regulated by the brain-specific microRNA, miR-124. This design forces the virus to strongly self-suppress in healthy neural tissue, solving a major safety concern for ZIKV-based therapies. ZIKV-miR124T is shown to be a potent oncolytic agent against malignant glioma while also serving as a highly effective, safe live-attenuated vaccine against ZIKV infection, reducing vertical transmission to the fetus. Our work provides a strong demonstration of utilizing microRNA regulation to achieve precise viral tropism and attenuation, offering a valuable, generalizable strategy for the development of safer and more effective viral therapies and vaccines against neurotropic pathogens.

## INTRODUCTION

Zika virus (ZIKV), a member of the *Flaviviridae* family, has transitioned from an obscure pathogen to a significant global health threat. The large-scale epidemics in the Americas (2015-2016) revealed ZIKV’s previously unrecognized pathogenic potential, most notably its causal association with severe congenital abnormalities such as fetal microcephaly, collectively termed Congenital Zika Syndrome (CZS) (1, 2). Further studies indicate that this pathogenic potential is largely attributable to ZIKV’s strong neurotropism, which underlies its ability to preferentially infect human neural progenitor cells (NPCs) in the developing brain and disrupt their proliferation and differentiation (3). In adults, ZIKV infection has also been associated with serious neurological complications, including Guillain-Barré syndrome (GBS) (4). These observations highlight the urgent need for a safe and effective ZIKV vaccine.

Among available vaccine platforms, live-attenuated vaccines (LAVs) have a long and successful record against several flaviviruses, including yellow fever virus (YFV) and Japanese encephalitis virus (JEV). The YF-17D vaccine, developed in the 1930s through extensive serial passage in chicken embryos, has been safely used for more than 80 years and remains the only widely deployed yellow fever vaccine (5). It induces strong and durable immunity, with a single dose providing lifelong protection in over 95% of recipients (6). Similarly, the JEV SA14-14-2 vaccine, derived from a wild-type strain through attenuation in cell culture, has been widely used in Asia since the 1980s and has proven to be highly safe and immunogenic in both children and adults (7). The success of these LAVs provides valuable precedents for rational vaccine design for ZIKV. Thus, following the recent large-scale epidemics, multiple strategies have been explored to attenuate ZIKV. For instance, by deleting specific sequences within the 3′ UTR of ZIKV, Shi et al. developed a LAV candidate (ZIKV-LAV) that is safe and elicits robust immune responses in mice following peripheral administration (8). In addition, insertions into the 5′ UTR or introduction of mutations within the viral ORF have also been shown to effectively attenuate ZIKV by restricting replication while maintaining immunogenicity, further supporting their potential as LAV candidates (8–10).

Beyond vaccine development, attenuated ZIKV strains have also attracted considerable interest as potential oncolytic agents, particularly for the treatment of glioma—the most common and aggressive primary brain tumor. Glioblastoma stem cells (GSCs), a subpopulation of glioma cells with self-renewal and tumor-initiating capacity, play critical roles in tumor progression, recurrence, and therapeutic resistance and have been well recognized as a major therapeutic target for glioma (11–13). Inspired by the shared biological similarities between NPCs and GSCs, including common stem cell markers and the capacity for self-renewal and differentiation, ZIKV has been explored as an oncolytic virus for targeting gliomas (14). We and others have shown that attenuated ZIKV strains can effectively suppress glioma growth in animal models by preferentially targeting GSCs, showing great potential of being oncolytic virus candidates (15–18). However, these previously developed candidates rely on global attenuation mechanisms, such as UTR deletions, and therefore lack tissue specificity. As a result, when these viruses are administered directly into the brain for glioma treatment, the risk of off-target infection in normal neuronal tissues cannot be fully eliminated.

MicroRNAs (miRNAs) are small non-coding RNAs that regulate gene expression post-transcriptionally by pairing with complementary sequences in target mRNAs, leading to their degradation or translational repression via the RNA-induced silencing complex (RISC) (19). Importantly, the expression of some miRNAs is highly tissue-specific or tissue-enriched. For instance, a panel of brain-specific or brain-enriched miRNAs, including miR-9-5p, −124-3p, and 219, has been identified (20). The highly specific expression profiles of certain miRNAs have been leveraged for viral engineering: by introducing cognate miRNA target sequences into a viral genome, the virus can be engineered to restrict its replication to corresponding miRNA-specific or enriched tissues, thereby replicating only in tissues where the specific miRNA is absent or minimally expressed (21, 22). Several engineered flaviviruses incorporating brain-specific miRNA targets have shown markedly reduced neuropathogenicity (23, 24). Moreover, recent studies have shown that certain tissue-specific or enriched miRNAs are substantially downregulated or lost in tumors derived from those tissues (25). This differential expression has also been exploited to enhance the safety of miRNA-regulated oncolytic viruses (26–29).

In this study, we identified miR-124 as a brain-specific miRNA that is highly expressed in NPCs and neurons, but nearly absent in GSCs and DGCs. Guided by this expression pattern, we engineered a recombinant ZIKV containing a miR-124 target sequence (miR-124T) and performed extensive *in vitro* and *in vivo* characterization. Our results show that ZIKV-miR124T displays profound neuroattenuation across multiple mouse models while eliciting strong protective immunity. Importantly, ZIKV-miR124T retains potent oncolytic efficacy against glioma, with a significantly superior safety profile compared with previous non-tissue-specific live-attenuated ZIKV candidates. Collectively, our findings provide a strong proof of concept for a rational, miRNA-guided approach to generate a next-generation ZIKV platform with dual potential as a safe LAV and a highly selective oncolytic virus.

## RESULTS

### Rational design and characterization of the miRNA-124 targeted recombinant ZIKV

**Fig 1:**
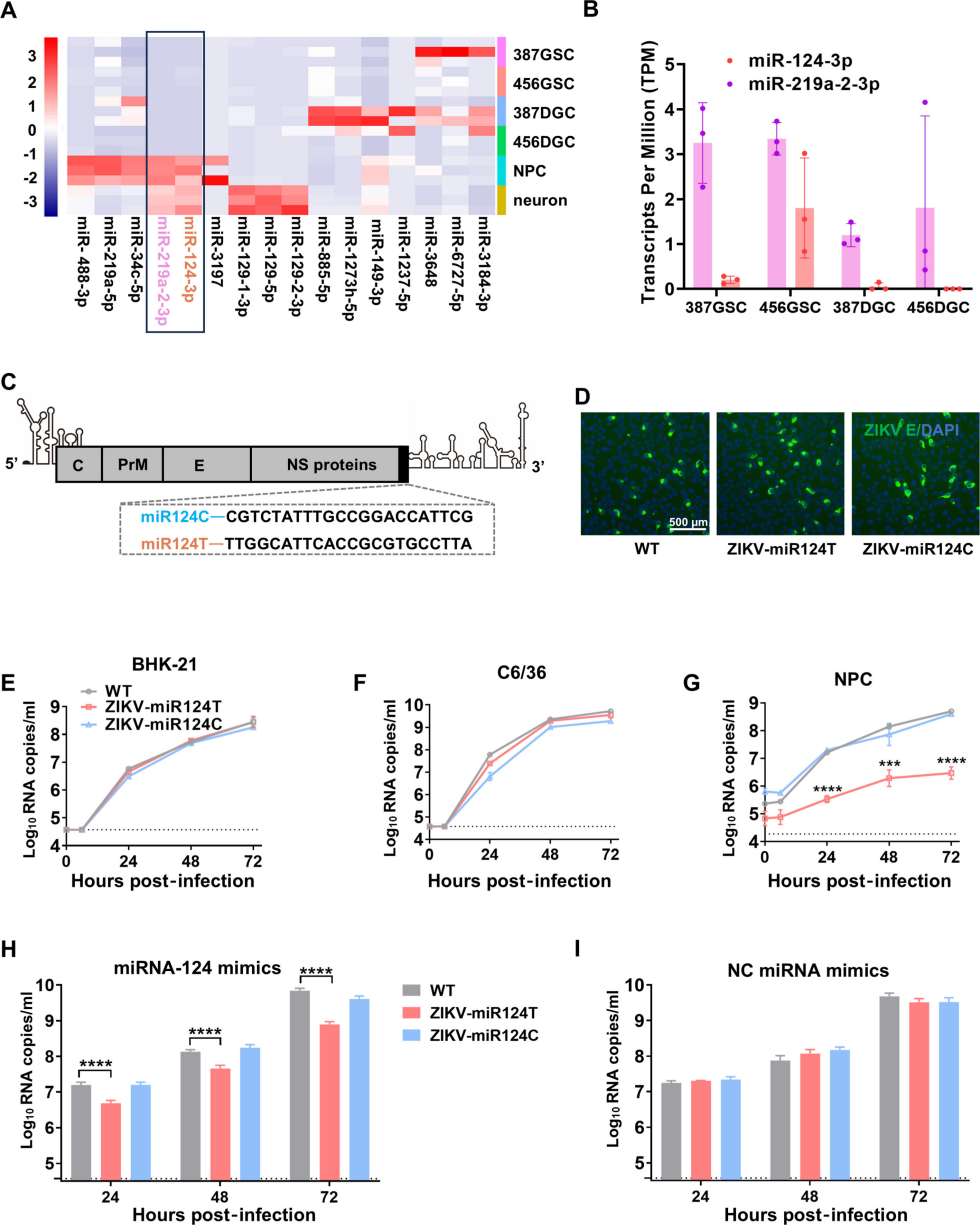
Rational design and characterization of miR-124-targeted recombinant ZIKV. (**A**) Heatmap showing the differential expression of brain-specific and enriched microRNAs between glioma cells (glioma stem cells [387GSC/456GSC] and differentiated glioma stem cells [387DGC/456DGC]) and neural cells (neural progenitor cells [NPCs] and neurons). Brain-specific and enriched microRNAs (miRNAs) were screened using the miRNATissueAtlas 2025 platform. MiRNA expression profiles in GSCs, DGCs, NPCs, and neurons were identified by small RNA sequencing. Data were row-standardized (Z-score): blue indicates low expression, white indicates mean expression, and red indicates high expression. The boxed region highlights miRNAs highly expressed in NPCs and neurons but minimally expressed in GSCs and DGCs. (**B**) Expression comparison of miR-124-3p and miR-219a-2-3p in GSCs and DGCs. (**C**) Schematic representation of the recombinant ZIKV genome incorporating the miR-124 target sequence (ZIKV-miR124T) or a scrambled control sequence (ZIKV-miR124C). (**D**) Immunostaining of the ZIKV envelope (E) protein in BHK-21 cells infected with the WT, ZIKV-miR124T, or ZIKV-miR124C viruses. (**E–G**) Growth kinetics of WT, ZIKV-miR124T, and ZIKV-miR124C in BHK-21 (**E**), C6/36 (**F**), and NPCs (**G**). (**H and I**) Antiviral activity of miR-124 mimics against ZIKV-miR124T. Vero cells were transfected with miR-124 or negative-control (NC) mimics and infected 6 h later with WT, ZIKV-miR124T, or ZIKV-miR124C at an MOI of 0.01. Viral RNA in culture supernatants was quantified by RT-qPCR at 24, 48, and 72 h post-infection. Data are presented as mean ± SD. Student’s *t*-test was used for statistical analysis. ***, *P* < 0.001; ****P < 0.0001.

To exploit the tissue-specific expression of miRNAs for the construction of a neuron-detargeted recombinant ZIKV, we first screened for brain-specific and brain-enriched miRNAs using the latest miRNATissueAtlas 2025 platform (20). Using filtering thresholds of average expression ≥100 reads per million (RPM) and a tissue specificity index (TSI) ≥0.75, we identified 26 brain-specific, highly expressed miRNAs (Table 1). To ensure that the recombinant ZIKV would not be attenuated in glioma cells, we focused on miRNAs that are abundant in normal neural cells but absent or minimally expressed in the target tumor cells. Accordingly, we performed small RNA sequencing (Fig. S1) and compared the expression of these 26 miRNAs between normal neural cells (NPCs and neurons) and glioma cells (GSCs and DGCs). As shown in Fig. 1A, 6 of the 26 brain-specific, highly expressed miRNAs were detected in our sequencing data set. Among them, miR-124-3p and miR-219a-2-3p were highly enriched in both NPCs and neurons while remaining nearly undetectable in GSCs and DGCs (Fig. 1A). Notably, miR-124-3p showed even lower expression in glioma cells than miR-219a-2-3p (Fig. 1B). Consequently, we selected the complementary target sequence of miR-124-3p (miR-124T) for incorporation into the ZIKV genome to achieve selective attenuation in normal neural tissues.

**TABLE 1:** Brain-specific and brain-enriched microRNAs

MicroRNA^*a*^	Avg expression	TSI
hsa-miR-124-3p	7344.6592	0.793
hsa-miR-3184-3p	2716.9075	0.9859
hsa-miR-2861	1129.5286	0.9336
hsa-miR-219a-2-3p	1056.5559	0.8928
hsa-miR-149-3p	1022.2205	0.9329
hsa-miR-6803-5p	925.5127	0.9691
hsa-miR-1237-5p	794.278	0.9738
hsa-miR-129-5p	557.2038	0.7593
hsa-miR-3197	505.3763	0.9708
hsa-miR-6126	500.9335	0.9663
hsa-miR-219a-5p	445.7605	0.7772
hsa-miR-4632-5p	443.6065	0.9739
hsa-miR-6131	414.6441	0.8701
hsa-miR-129-2-3p	370.1896	0.754
hsa-miR-4271	343.1552	0.9809
hsa-miR-6727-5p	324.4713	0.9667
hsa-miR-6771-5p	300.1128	0.9709
hsa-miR-6756-5p	237.7932	0.9793
hsa-miR-1273h-5p	200.0992	0.8622
hsa-miR-4309	179.3453	0.9716
hsa-miR-488-3p	177.8843	0.779
hsa-miR-129-1-3p	174.9412	0.8003
hsa-miR-6752-5p	166.9258	0.9794
hsa-miR-885-5p	142.5589	0.811
hsa-miR-3648	119.0083	0.88
hsa-miR-34c-5p	112.0561	0.7609

^*a*^ The screen was conducted using the latest miRNATissueAtlas 2025 platform. A set of 26 highly expressed, brain-specific miRNAs was identified by applying filtering cutoffs of average expression (avg_expression) ≥100 reads per million (RPM) and a tissue specificity index (TSI) ≥ 0.75.

Because the ZIKV open reading frame (ORF) is translated as a single polyprotein and the 5′ and 3′ untranslated regions (UTRs) play essential roles in viral replication, we inserted the miR-124T sequence immediately downstream of the ORF stop codon within the proximal 3′ UTR, a region chosen to minimize disruption of translation or genome architecture (Fig. 1C). Using our previously developed full-length infectious ZIKV clone as a backbone, we generated two recombinant viruses by inserting either the miR-124T sequence or a length-matched scrambled control sequence (miR-124C) at this locus (Fig. 1C). The resulting recombinant viruses, ZIKV-miR124T and ZIKV-miR124C, were rescued via *in vitro*-transcribed RNA transfection into BHK-21 cells (Fig. 1D). To assess the genetic stability, ZIKV-miR124T was serially passaged 20 times in BHK-21 or Vero cells. Full-genome sequencing of the passaged virus revealed no deletion and nucleotide substitutions in the inserted sequence, confirming the high genetic stability of the recombinant viruses (Fig. S2).

We next examined whether insertion of the miR-124T sequence attenuated viral replication in miR-124-expressing cells. Growth curve analysis by RT-qPCR showed that WT ZIKV, ZIKV-miR124T, and ZIKV-miR124C replicated similarly in non-neural cells, including BHK-21 and C6/36 cells (Fig. 1E and F), indicating that insertion of the miR-124T sequence did not impair overall viral replication competence. In contrast, in NPCs that express high levels of miR-124, replication of ZIKV-miR124T was markedly reduced compared with WT or ZIKV-miR124C (Fig. 1G). Infectious viral titers by plaque-forming assay showed similar results (Fig. S3).

To directly test whether this attenuation was mediated by miR-124-dependent silencing, we performed gain-of-function experiments. Vero cells were transfected with either a synthetic miR-124 mimic or a negative-control mimic and subsequently infected with the three viruses. As expected, production of ZIKV-miR124T was substantially reduced in the presence of miR-124 mimic, whereas replication of WT and ZIKV-miR124C remained unchanged (Fig. 1H). Meanwhile, none of the viruses were affected by the control mimic (Fig. 1I). Together, these data demonstrate that ZIKV-miR124T retains full replication competence in non-neural cells while undergoing precise miR-124-dependent attenuation in NPCs.

### ZIKV-miR124T shows an attenuated phenotype in adult mice

**Fig 2:**
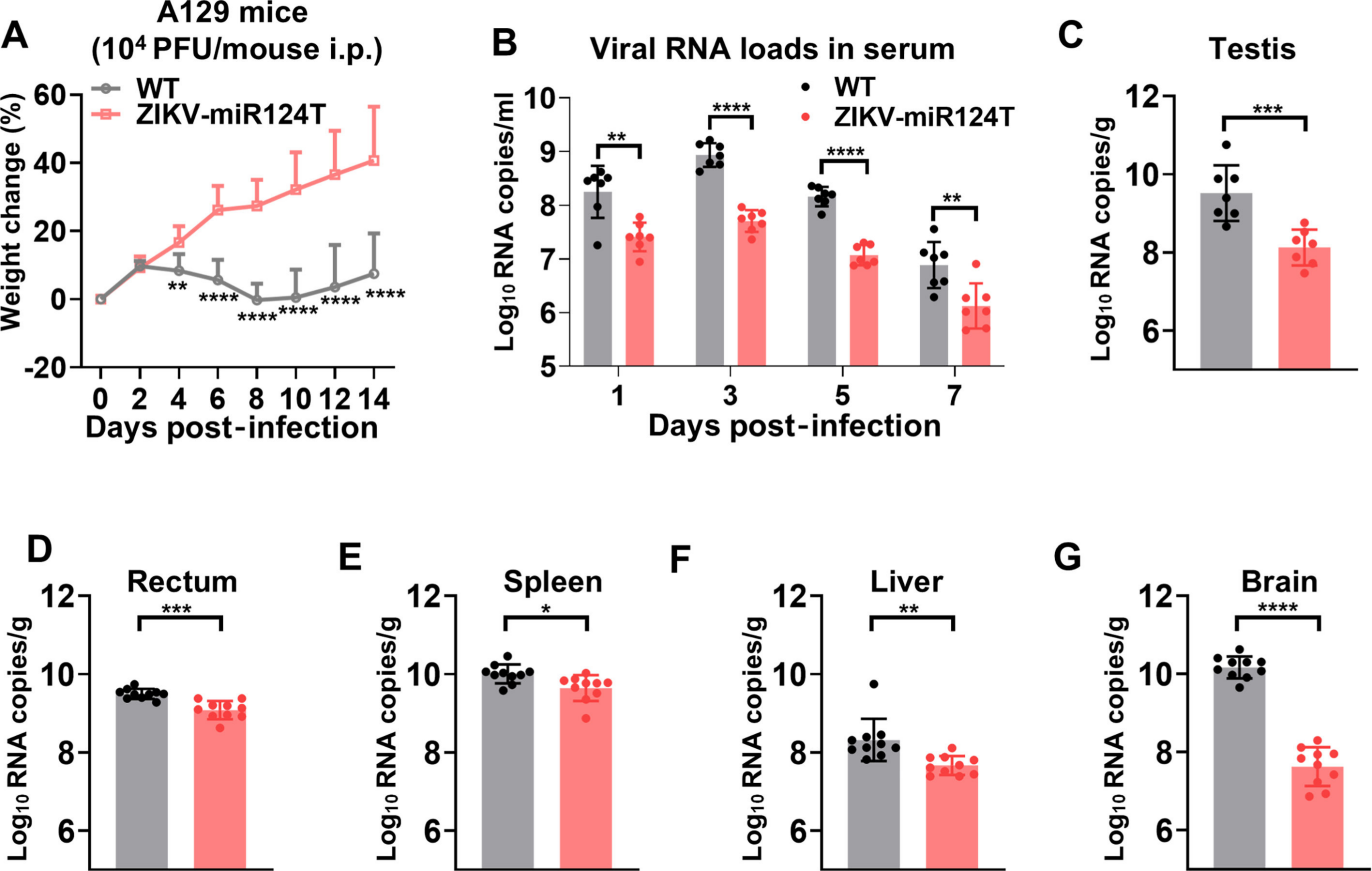
The attenuation phenotype of ZIKV-miR124T in adult A129 mice. Adult A129 mice were intraperitoneally (i.p.) infected with 10^4^ PFU of WT or ZIKV-miR124T. Body weight change (**A**) and viral RNA loads in serum (**B**) of infected mice (*n* = 7) were monitored at the indicated time points. (**C–G**) Viral RNA loads in selected tissues were detected on day 7 post-infection (dpi) (*n* = 10). Viral RNA loads were quantified by RT-qPCR. Data are presented as mean ± SD. Student’s *t*-tests were used for statistical analysis. *, *P* < 0.05; **, *P* < 0.01; ***, *P* < 0.001; ****, *P* < 0.0001.

To evaluate the attenuation of ZIKV-miR124T, we compared its virulence to that of the WT virus in adult A129 mice, which lack the IFN-α/β receptor. Following intraperitoneal injection with 10^4^ PFU of virus, WT-infected mice exhibited a transient decrease in body weight from 4 to 8 days post-infection (dpi), whereas mice in the ZIKV-miR124T group continued to gain weight throughout the experiment (Fig. 2A). Consistent with the clinical observations, ZIKV-miR124T infection resulted in significantly reduced viral RNA loads (~6–17-fold) in the serum compared to WT infection (Fig. 2B). Similarly, reduced viral RNA loads were also observed in peripheral organs of ZIKV-miR124T-infected mice compared to WT controls, including the testis (~25-fold), rectum (~2.6-fold), spleen (~2.3-fold), and liver (~4.5-fold) (Fig. 2C through F). Most notably, ZIKV-miR124T resulted in a more dramatic reduction in viral RNA loads in the brain (~350-fold lower) compared to WT (Fig. 2G). These data demonstrate that ZIKV-miR124T exhibits an attenuated phenotype in adult A129 mice, particularly in the brain.

### ZIKV-miR124T exhibits an attenuated phenotype in pregnant mice

**Fig 3:**
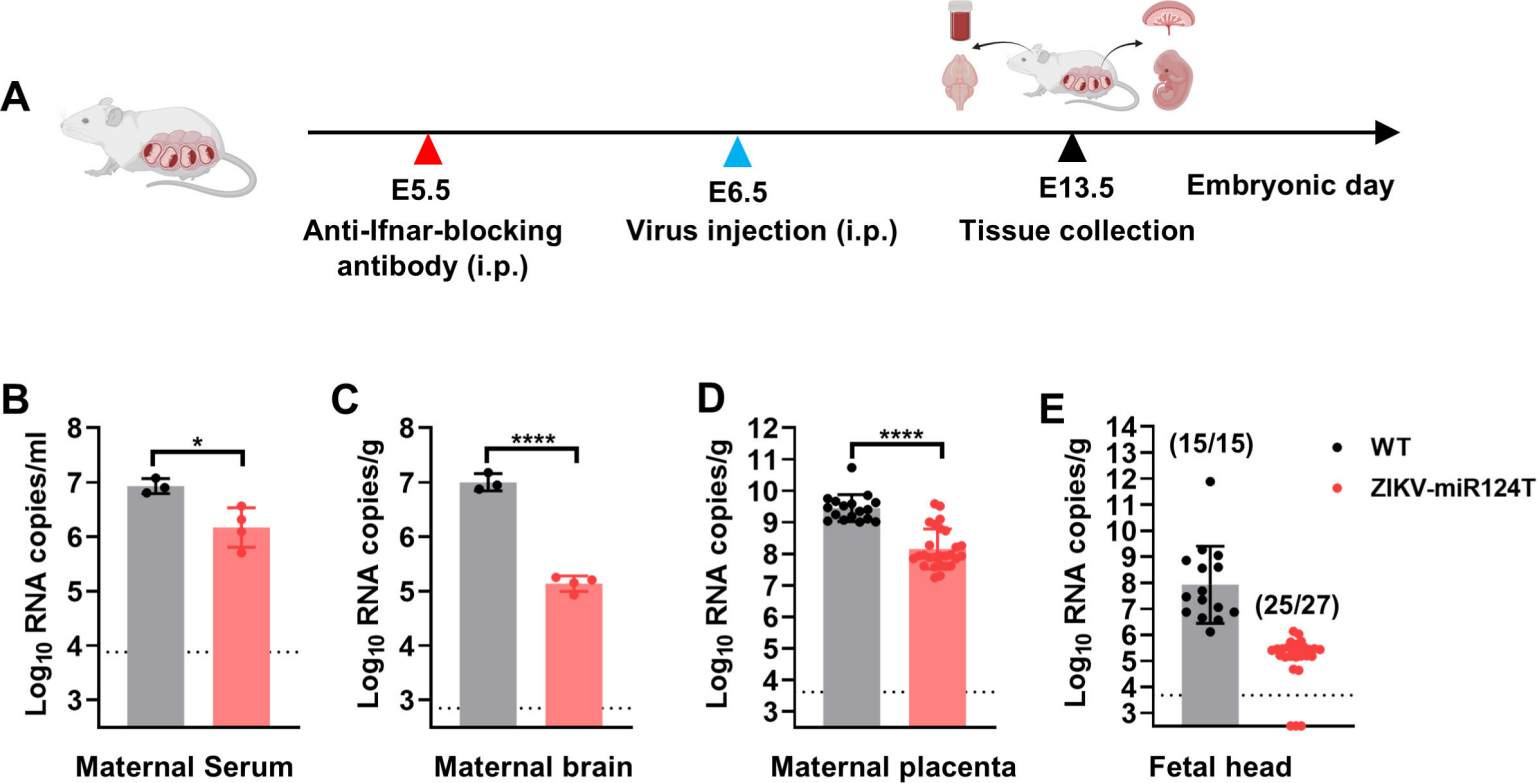
Decreased viral loads of ZIKV-miR124T in the placenta and fetal heads. (**A**) Experimental scheme. Pregnant mice were administered 2.0 mg of anti-Ifnar1-blocking antibody via intraperitoneal (i.p.) injection at embryonic day (E) 5.5, followed by i.p. infection with WT or ZIKV-miR124T at E6.5. (**B–E**) Viral RNA loads in maternal sera (**B**), maternal brains (**C**), placentas (**D**), and fetal heads (**E**) collected at E13.5. Viral RNA amounts were quantified by RT-qPCR (*n* = 3–4 dams per group). Data are presented as mean ± SD. Student’s *t*-test was used for statistical analysis. *, *P* < 0.05; ****, *P* < 0.0001. The dotted line indicates the limit of detection (LOD) based on Ct = 40. Samples with Ct values ≥ 40 are considered not detected (ND) and are plotted below the LOD. Numbers shown in parentheses above each group indicate the number of samples with detectable viral RNA above the LOD (detected/total, n/N).

We next evaluated the potential for vertical transmission of ZIKV-miR124T in a pregnant BALB/c mouse model (30). To permit efficient viral infection in immunocompetent dams, an anti-Ifnar1 blocking antibody was administered at embryonic day 5.5 (E5.5), followed by intraperitoneal viral challenge (Fig. 3A). As expected, WT infection resulted in high viral RNA loads in maternal serum and brain at E13.5, whereas these levels were significantly reduced in ZIKV-miR124T-infected dams (Fig. 3B and C). Consistent with this observation, viral RNA loads were also significantly lower in placentas from ZIKV-miR124T-infected dams compared to those from WT-infected dams (Fig. 3D). More importantly, while high viral RNA loads (~8.4 × 10⁷ copies/g) were detected in all fetal heads (15/15) from WT-infected dams, substantially lower levels (~1.9 × 10^5^ copies/g) were detected in 25 of 27 fetal heads from the ZIKV-miR124T group (Fig. 3E). In summary, ZIKV-miR124T demonstrates a significantly attenuated phenotype in the pregnant mouse model.

### ZIKV-miR124T exhibits significantly attenuated neurovirulence in neonatal mice

**Fig 4:**
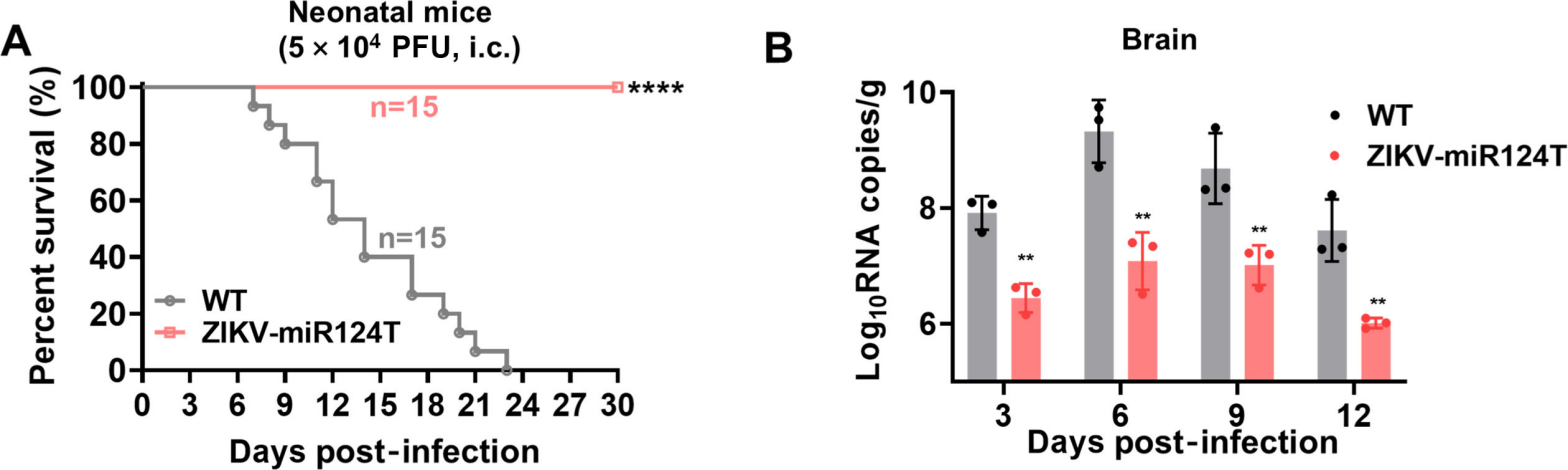
The ZIKV-miR124T exhibits attenuated neurovirulence in neonatal mice. (**A**) Survival curves in 1-day-old BALB/c neonatal mice injected intracerebrally (i.c.) with 5 × 10^4^ PFU of WT or ZIKV-miR124T (*n* = 15). Survival was analyzed using the log-rank test. ns, *P* > 0.05, ****P < 0.0001. (**B**) Viral RNA levels in brains of neonatal mice injected i.c. with 5 × 10^3^ PFU of WT or ZIKV-miR124T. Brains were collected on days 3, 6, 9, and 12 post-infection, and viral RNA was quantified by RT-qPCR. Data are presented as mean ± SD. Student’s *t*-test was used for statistical analysis. **, *P* < 0.01.

Having established that ZIKV-miR124T is attenuated in adult mice and exhibits restricted vertical transmission in pregnant dams, we next assessed its neurovirulence in the highly susceptible neonatal mouse model (31). One-day-old BALB/c neonatal mice were intracerebrally inoculated with equivalent doses of WT ZIKV or ZIKV-miR124T. Following inoculation, WT-infected mice rapidly developed severe neurological symptoms, including inactivity, motor weakness, and bilateral hind-limb paralysis, and all succumbed to infection within 23 days. In contrast, all ZIKV-miR124T-infected mice survived without exhibiting any clinical signs of disease throughout the observation period (Fig. 4A). Furthermore, viral RNA loads in the brains of ZIKV-miR124T-infected mice were significantly reduced, showing a 30- to 180-fold decrease compared with WT-infected mice from 3 to 12 dpi (Fig. 4B). These results demonstrate that incorporation of the miR-124 target sequence effectively attenuates ZIKV neurovirulence in neonatal mice.

### ZIKV-miR124T maintains potent oncolytic effects and exhibits ideal glioma selectivity

**Fig 5:**
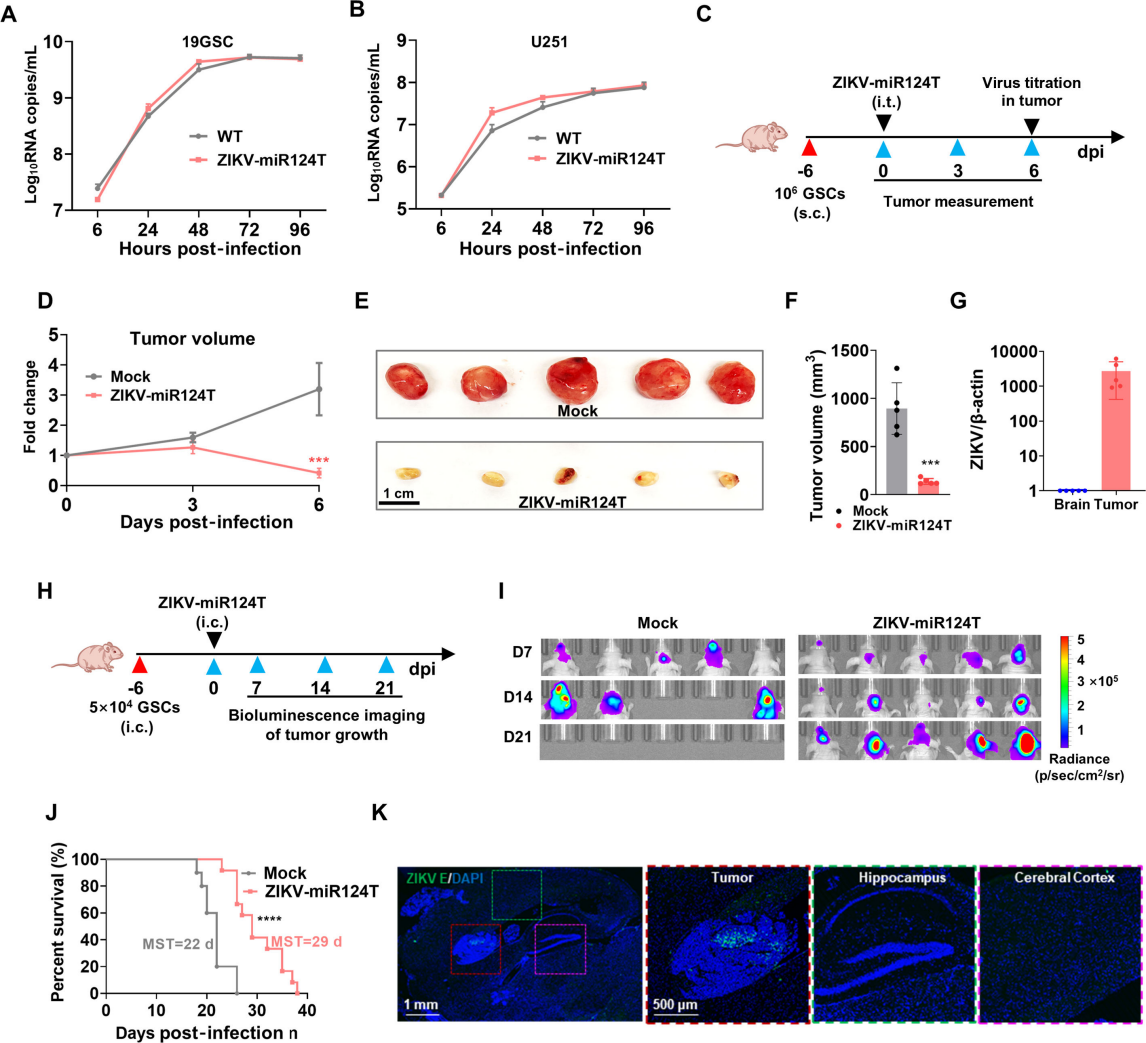
ZIKV-miR124T maintains potent oncolytic effects and exhibits ideal selectivity for glioma. (**A and B**) Replication kinetics of WT ZIKV and ZIKV-miR124T in glioma stem cells (19GSC, MOI = 1) and glioma cells (U251, MOI = 0.1). Cell culture supernatants were collected at the indicated time points, and viral RNA levels were quantified by RT-qPCR. (**C–G**) Oncolytic activity in a heterotopic xenograft glioma model. Three-week-old BALB/c nude mice were subcutaneously (s.c.) implanted with 10⁶ 19GSCs. On day 6 post-implantation, tumors were intratumorally (i.t.) injected with 10⁴ PFU of ZIKV-miR124T or phosphate-buffered saline (PBS; mock control). The experimental scheme depicts the tumor implantation and intratumoral treatment schedule; dpi, days post-injection. (**D**) Tumor volumes on days 3 and 6 post-i.t. injection, shown as fold change relative to day 0. (**E and F**) Subcutaneous tumors were surgically excised and photographed (**E**), and tumor volumes were measured (**F**) 6 days after i.t. virus administration. (**G**) Viral RNA levels (ZIKV/β-actin normalized) in tumor and brain tissues collected 6 days post-i.t. injection. (**H–K**) Oncolytic activity in an orthotopic glioma model. Three-week-old BALB/c nude mice were intracerebrally (i.c.) implanted with 5 × 10⁴ luciferase-labeled 19GSCs. On day 6 post-implantation, mice received an intracerebral injection of 10⁴ PFU of ZIKV-miR124T or PBS (mock control). (**H**) Experimental scheme showing intracranial implantation and treatment schedule. (**I**) *In vivo* bioluminescence imaging of tumor growth on days 7, 14, and 21 post-injection. (**J**) Survival curves of ZIKV-miR124T- and mock-treated mice (*n* = 10). Median survival time, MST. (**K**) Representative immunofluorescence images of tumor-bearing brain tissues showing ZIKV envelope (E) protein (green) and DAPI (blue). Magnified insets from the tumor, hippocampus, and cortex illustrate the selective localization of ZIKV E protein within the tumor mass. Data are presented as mean ± SD. Log-rank tests were used for survival analyses. Student’s *t*-test was used for two-group comparisons. ***, *P* < 0.001; ****, *P* < 0.0001.

Having confirmed its markedly attenuated neurovirulence, we next examined whether ZIKV-miR124T retains its infectivity and oncolytic activity against glioma. We first analyzed the replication kinetics of ZIKV-miR124T in glioma cells, including 19GSC and U251. As expected, although ZIKV-miR124T replicated significantly less efficiently in NPCs (Fig. 1G), its replication in both 19GSC and U251 cells was comparable to that of the WT virus (Fig. 5A and B). We next evaluated oncolytic efficacy in mice using a heterotopic xenograft model generated by subcutaneous implantation of 19GSC cells (Fig. 5C) (32). On day 6 post-implantation, tumors were injected intratumorally with 10⁵ PFU of ZIKV-miR124T or PBS (mock). Assessment of tumor growth revealed that ZIKV-miR124T induced significant tumor regression (Fig. 5D through F). Furthermore, viral RNA was only detected in tumors but absent in the brain (Fig. 5G), indicating that ZIKV-miR124T remained confined to the tumor site and did not invade the brain.

After confirming the ideal tumor selectivity of ZIKV-miR124T in the heterotopic model (Fig. 5G), we next established an orthotopic xenograft model to evaluate its therapeutic efficacy and safety within the native brain microenvironment (32). The orthotopic model was generated by intracerebral implantation of luciferase-labeled 19GSC cells (Fig. 5H). On day 6 post-implantation, tumor-bearing mice were intracerebrally injected with 10⁴ PFU of ZIKV-miR124T or PBS (mock). ZIKV-miR124T demonstrated significant antitumor activity in this model, resulting in reduced glioma growth in a subset of treated mice (Fig. 5I) and a significant extension of median survival time of tumor-bearing mice from 22 to 29 days (Fig. 5J). To further assess safety following intracerebral administration, brain tissues were collected on day 21 post-treatment for viral distribution analysis. Immunofluorescence (IF) staining for viral proteins revealed that ZIKV-miR124T was strictly confined to the tumor regions, with no detectable signals in surrounding normal brain tissue (Fig. 5K). Collectively, these results demonstrate that ZIKV-miR124T maintains potent oncolytic activity and exhibits excellent glioma selectivity, even after direct intracranial administration.

### ZIKV-miR124T exhibits markedly improved safety while maintaining oncolytic efficacy comparable to ZIKV-LAV

**Fig 6:**
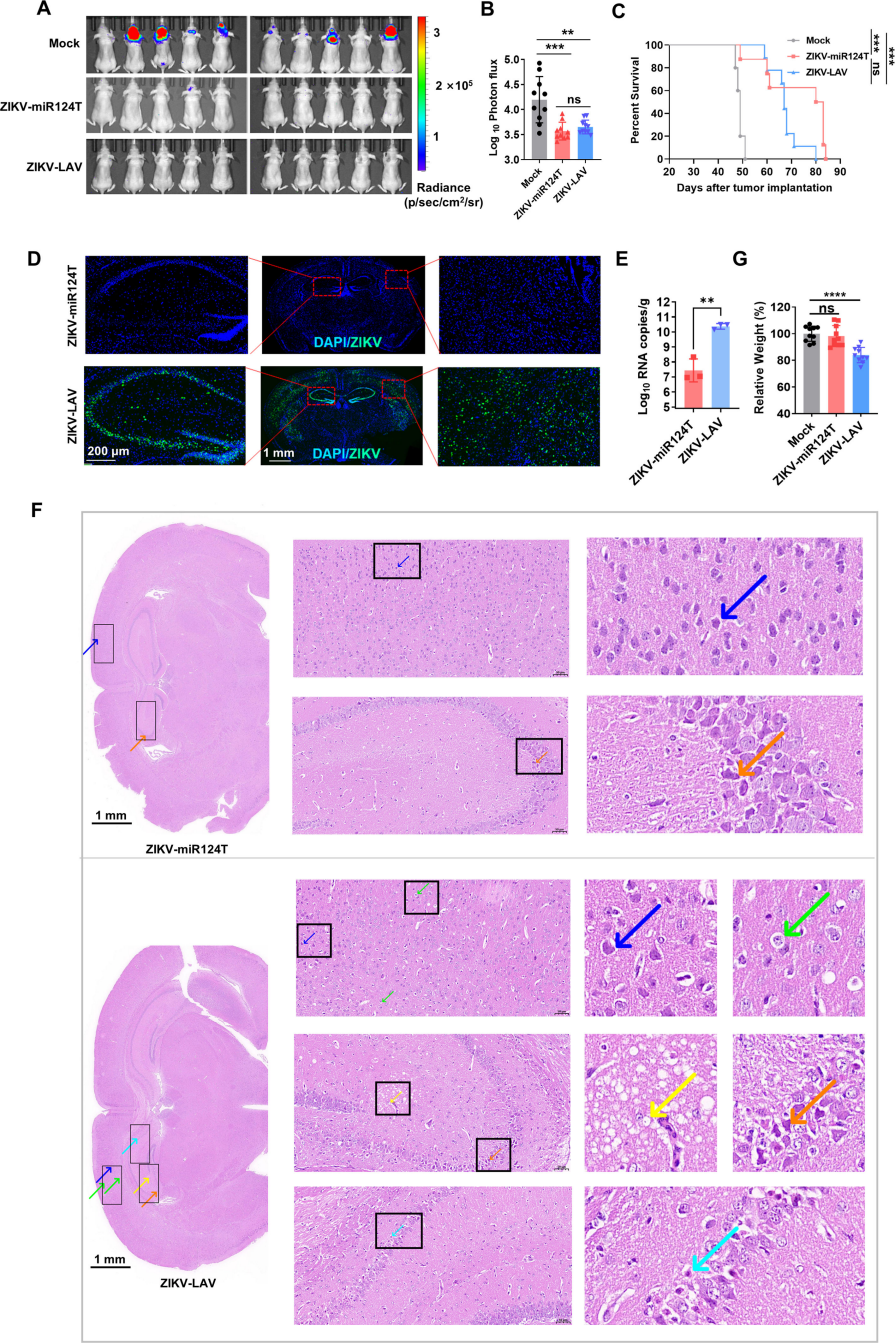
ZIKV-miR124T exhibits improved safety and comparable oncolytic efficacy relative to ZIKV-LAV. Three-week-old BALB/c nude mice were intracerebrally (i.c.) transplanted with 10^4^ PFU of ZIKV-LAV, ZIKV-miR124T, or RPMI 1640 (mock) mixed with 5 × 10^4^ luciferase-labeled 387GSCs. (**A**) *In vivo* bioluminescence imaging of tumor growth on day 21 post-implantation. (**B**) Quantification of photon flux from mouse brains. Statistical significance was analyzed by unpaired Student’s *t*-test. ns, *P >* 0.05; *** *P* < 0.001. (**C**) Survival curves of mice treated with ZIKV-miR124T, ZIKV-LAV, or mock. Statistical significance was assessed by the log-rank test. ns, *P* > 0.05; ***, *P <* 0.001. (**D–F**) Safety and biodistribution analysis on day 21 post-implantation. (**D**) Representative immunofluorescence images of brain sections from ZIKV-LAV- or ZIKV-miR124T-treated mice showing ZIKV E protein (green) and DAPI (blue). (**E**) The viral RNA copies in normal brain tissues were quantified by RT-qPCR. Statistical significance was analyzed by unpaired Student’s *t*-test. **, *P* < 0.01. (**F**) Hematoxylin and eosin (H&E) staining of brain sections from ZIKV-LAV- or ZIKV-miR124T-treated mice. Orange arrows indicate pyramidal neurons with cytoplasmic shrinkage, cyan arrows indicate pyramidal neuron necrosis, yellow arrows indicate perineuronal/neuropil vacuolation, green arrows indicate neuronal degeneration, and blue arrows denote shrunken neurons with condensed cytoplasm. (**G**) Relative body weight change on day 21 post-implantation, normalized to the mock control group. Statistical significance was analyzed by unpaired Student’s *t*-test. ns, *P* > 0.05; ***, *P* < 0.001.

To further evaluate the oncolytic efficacy and safety profile of ZIKV-miR124T, we directly compared it with a previously characterized live-attenuated ZIKV strain (ZIKV-LAV) (8, 16). 387GSC stably expressing firefly luciferase were co-implanted with ZIKV-miR124T or ZIKV-LAV into the brains of BALB/c nude mice, and tumor growth was monitored by measuring bioluminescence signals. As shown in Fig. 6A through C, both ZIKV-miR124T and ZIKV-LAV effectively inhibited glioma growth and significantly prolonged survival in tumor-bearing mice. Notably, mice treated with ZIKV-miR124T exhibited a longer median survival than those treated with ZIKV-LAV, although the difference did not reach statistical significance.

For safety evaluation, brain tissues were collected on day 21 post-treatment for viral distribution and replication analysis. Immunofluorescence (IF) staining showed widespread distribution of viral proteins throughout the brains of ZIKV-LAV-treated mice, predominantly in the hippocampal and cortical regions (Fig. 6D). In contrast, ZIKV viral protein was barely detectable in the whole brain following ZIKV-miR124T infection (Fig. 6D). Consistent with these IF staining results, viral RNA quantification showed that ZIKV-miR124T viral RNA levels in normal brain tissues were nearly 1,000-fold lower than those in ZIKV-LAV-treated mice (Fig. 6E). Further histopathological examination indicated that intracerebral administration of ZIKV-LAV induced minimal yet detectable neuronal alterations in normal brain (Fig. 6F). In the hippocampus, occasional pyramidal neurons in the CA3 region exhibited cytoplasmic shrinkage (orange arrows), characterized by reduced cell volume, irregular cell contours, indistinct nuclear-cytoplasmic boundaries, and increased staining intensity. In the CA1, CA2, and CA3 regions, scattered pyramidal neurons displayed eosinophilic neuronal necrosis (cyan arrows) with marked nuclear pyknosis, fragmentation, and dissolution. Mild perineuronal and neuropil vacuolation was occasionally observed surrounding the dentate gyrus (yellow arrows). In the cortex, a small number of neurons showed degenerative changes (green arrows), including cell swelling and pale, loose cytoplasm, whereas others showed neuronal shrinkage (blue arrows) with reduced soma size, irregular morphology, and cytoplasmic hyperchromasia. By contrast, mice treated with ZIKV-miR124T showed fewer pathological changes. Only occasional pyramidal neurons in the CA3 region presented mild cytoplasmic shrinkage (orange arrows), and rare shrunken neurons were observed in the cortex (blue arrows). Importantly, no neuronal necrosis or vacuolation was detected. Regarding systemic safety, mice treated with ZIKV-LAV exhibited significant body weight loss on 21 dpi, whereas those receiving ZIKV-miR124T maintained stable body weight comparable to that of the mock-treated group (Fig. 6G). These data demonstrate that incorporation of miR-124T substantially reduces off-target neurotoxicity and further improves the safety profile of ZIKV-based oncolytic therapy, while effectively maintaining its therapeutic efficacy.

### The ZIKV-miR124T virus elicits protective immunity in A129 mice

**Fig 7:**
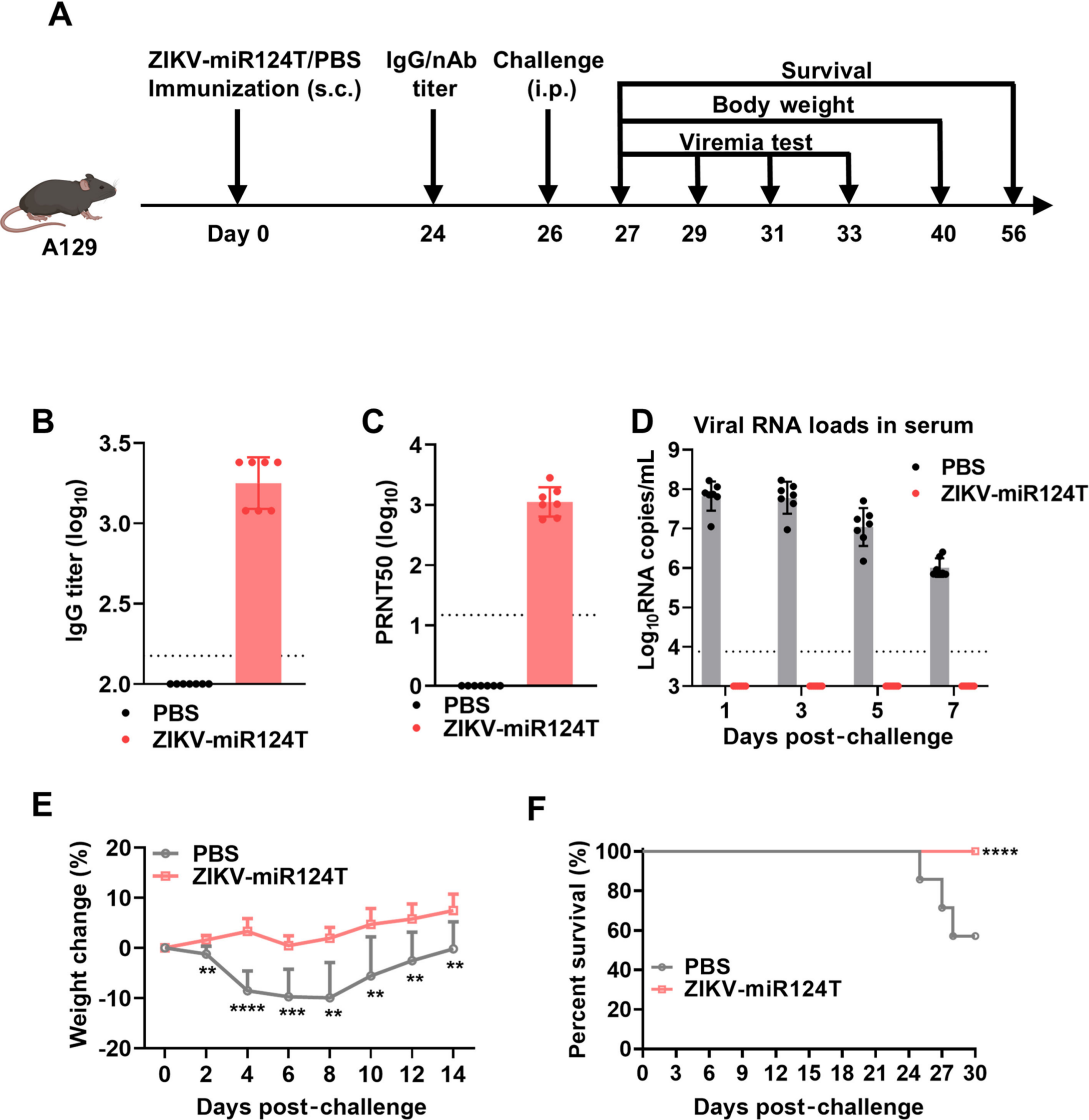
ZIKV-miR124T elicits robust neutralizing immunity and provides protection in A129 mice. (**A**) Experimental scheme. Four-week-old A129 mice (*n* = 7) were immunized subcutaneously (s.c.) with 10⁴ PFU of ZIKV-miR124T or PBS. (**B and C**) Humoral immune responses on day 24 post-immunization. (**B**) Serum IgG titers measured by ELISA. (**C**) Neutralizing antibody (nAb) titers determined by PRNT₅₀ (plaque reduction neutralization test 50%). (**D–F**) Protective efficacy against WT ZIKV challenge. On day 26 post-immunization, mice were challenged intraperitoneally (i.p.) with 10⁵ PFU of WT ZIKV. Challenged mice were monitored for viremia (**D**), weight loss (**E**), and survival (**F**). Data are presented as mean ± SD. The dotted line indicates the limit of detection based on Ct = 40. Samples with Ct values ≥ 40 are considered not detected (ND) and are plotted below the LOD. Log-rank tests were used for survival analysis. Student’s *t*-test was used for two-group comparisons. **, *P* < 0.01; ***, *P* < 0.001; ****, *P* < 0.0001.

Given the high degree of attenuation observed for ZIKV-miR124T in multiple mouse models, we next evaluated its potential as a LAV vaccine candidate in A129 mice. Groups of A129 mice were subcutaneously immunized with 10⁴ PFU of ZIKV-miR124T or PBS as a control. On day 26 post-immunization, serum antibody responses were assessed using ELISA and PRNT₅₀ assays (Fig. 7A). As shown in Fig. 7B and C, all ZIKV-miR124T-immunized mice developed robust IgG and neutralizing antibody responses. To evaluate protective efficacy, immunized mice were challenged with 10⁵ PFU of WT ZIKV on day 26 post-immunization. As expected, all PBS-immunized control mice developed high viral RNA loads in the serum, whereas viral RNA was undetectable in all ZIKV-miR124T-immunized mice (Fig. 7D). Meanwhile, all PBS-immunized mice exhibited rapid and progressive weight loss beginning immediately after challenge and continuing until day 8. In contrast, ZIKV-miR124T-immunized mice displayed only a transient, mild weight loss on day 6, after which their body weight rapidly returned to baseline (Fig. 7E). Crucially, over 40% of PBS-immunized mice succumbed to infection, whereas 100% of ZIKV-miR124T-immunized mice survived without exhibiting any clinical signs of disease throughout the monitoring period (Fig. 7F). Collectively, these results demonstrate that ZIKV-miR124T induces potent antibody responses that confer significant protection against ZIKV challenge in A129 mice.

### ZIKV-miR124T induces protective immunity in pregnant mice

**Fig 8:**
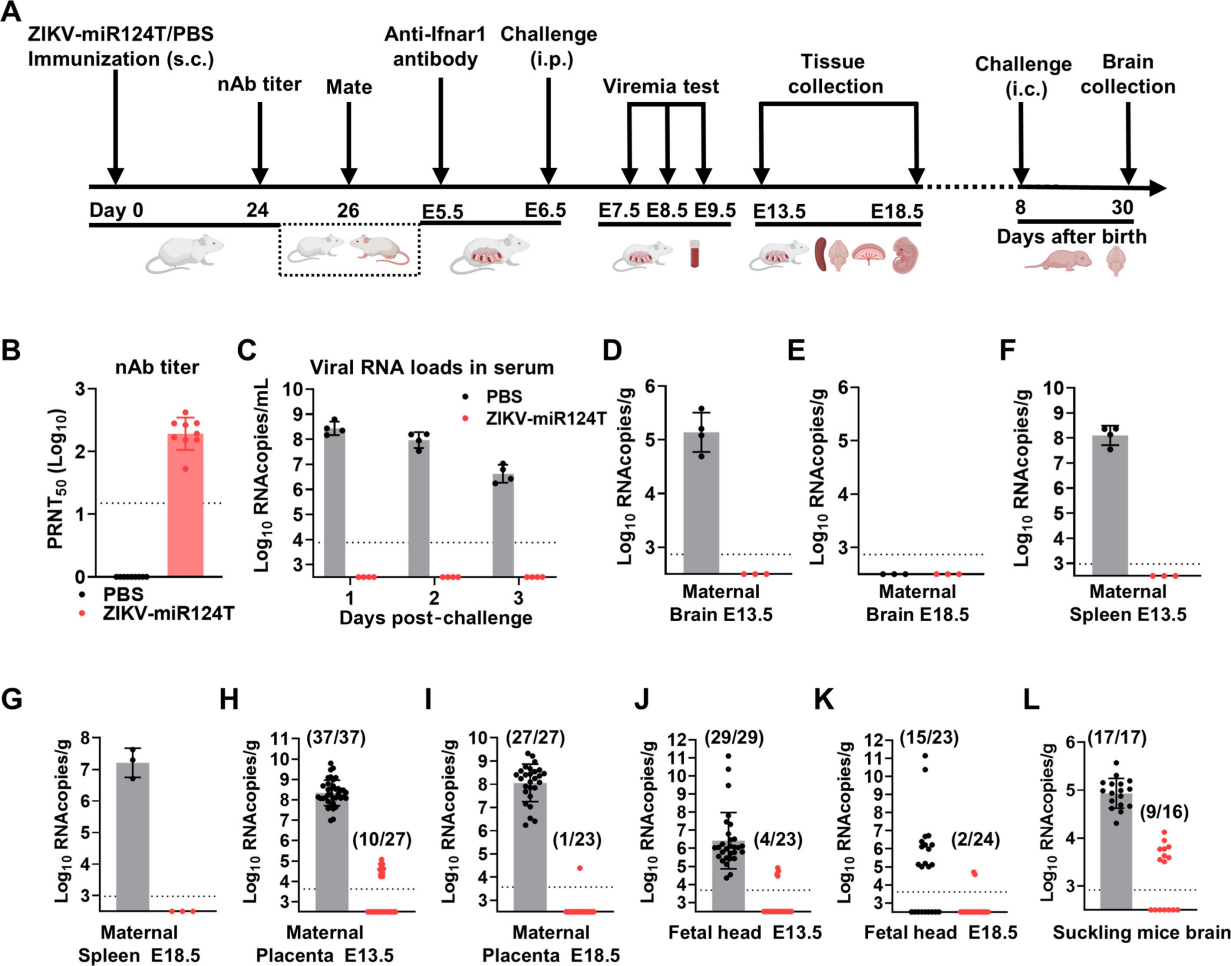
ZIKV-miR124T immunization protects against vertical transmission and post-natal ZIKV challenge. (**A**) Experimental scheme. Six-week-old BALB/c female mice (*n* = 9) were immunized subcutaneously (s.c.) with 10⁶ PFU of ZIKV-miR124T or PBS. At day 26 post-immunization, immunized females were mated with BALB/c males. Pregnant dams were administered 2.0 mg of anti-Ifnar1-blocking antibody intraperitoneally (i.p.) at embryonic day 5.5 (E5.5), followed by subcutaneous challenge with 10⁵ PFU of WT ZIKV at E6.5. (**B**) Neutralizing antibody responses. Serum neutralizing antibody (nAb) titers were measured on day 24 post-immunization using the PRNT₅₀ assay. (**C**) Maternal viremia. Viral RNA loads in maternal sera collected from E7.5 to E9.5 post-challenge were quantified by RT-qPCR. (**D–K**) Viral RNA loads in maternal and fetal tissues. Viral RNA levels in maternal brains and spleens, as well as placentas and fetal heads, at E13.5 (**D, F, H, J**) and E18.5 (**E, G, I, K**) were quantified by RT-qPCR. (**L**) Post-natal challenge. Suckling mice born to ZIKV-miR124T-immunized or PBS-treated dams (non-immune controls) were intracerebrally (i.c.) challenged with 10^5^ PFU of WT on day 8 after birth. Viral RNA loads in the brain were determined by RT-qPCR on day 22 after infection. Data are shown as individual values. Summary bars (when shown) represent mean ± SD of detected samples only. The dotted line indicates the limit of detection (LOD) based on Ct = 40. Samples with Ct values ≥ 40 are considered not detected (ND) and are plotted below the LOD. Numbers shown in parentheses above each group indicate the number of samples with detectable viral RNA above the LOD (detected/total, n/N).

Because prevention of maternal-fetal transmission is a critical public health goal in ZIKV infection, we next evaluated the protective efficacy of ZIKV-miR124T using an established vertical-transmission mouse model (30, 33, 34) (Fig. 8A). Consistent with our previous results, ZIKV-miR124T induced strong neutralizing antibody responses on 24 dpi in all ZIKV-miR124T-immunized pregnant dams (Fig. 8B). Following intraperitoneal challenge, all PBS-immunized control dams developed high viral RNA loads in the serum on 1–3 dpi, whereas ZIKV-miR124T-immunized dams displayed no detectable viral RNA (Fig. 8C). This protection extended to maternal tissues: high levels of ZIKV RNA were detected in the brains and spleens of control dams at E13.5 and E18.5, whereas no viral RNA was detected in all ZIKV-miR124T-immunized dams (Fig. 8D through G).

As expected, high levels of viral RNA were detected in all placentas (37/37) from PBS-immunized dams at E13 and E18 (Fig. 8H and I), while viral RNA was only detected in a minority of placentas (10/27 and 1/23) from the ZIKV-miR124T-immunized animals, with significantly decreased viral RNA loads (Fig. 8H and I). Further examination of fetal heads at E13.5 and E18.5 also showed similar results (Fig. 8J and K).

Finally, to assess whether protective immunity was transferred to the offspring, 8-day-old suckling mice from ZIKV-miR124T- or PBS-immunized dams were intracerebrally challenged with 10⁵ PFU of WT ZIKV. By day 22 post-infection, high levels of viral RNA (~10^4.9^ copies/g) were detected in all brains (17/17) of offspring from PBS-immunized dams, whereas viral RNA was detected in only 9 of 16 offspring from ZIKV-miR124T-immunized dams with substantially lower levels (~10^3.7^ copies/g) (Fig. 8L). Taken together, ZIKV-miR124T elicits potent neutralizing antibody responses that provide significant protection against ZIKV infection in pregnant dams, developing fetuses, and suckling offspring.

## DISCUSSION

ZIKV’s intrinsic neurotropism presents a dual-edged property: it causes severe neuropathology while also providing a unique basis for targeting gliomas (35–37). To harness this potential safely, neurovirulence must be attenuated without severely impairing replication competence. In this study, we achieved this balance through a rational, miRNA-guided design: ZIKV-miR124T incorporates the target sequence of miR-124, a neuron-specific microRNA carefully selected for its abundant expression in normal neural tissues but minimal presence in gliomas and peripheral tissues. This precisely tuned regulatory element allows the virus to self-suppress in normal neural tissues while replicating efficiently in tumors and peripheral organs, thereby establishing a scientifically elegant dual-functional platform: safe as a live-attenuated vaccine and potent as an oncolytic virus.

The ZIKV-miR124T we developed in this study showed a significantly increased level of neuroattenuation. Previously, several attenuated ZIKV strains have been developed using different strategies. For instance, Shi et al. achieved ZIKV attenuation by deleting specific sequences within the 3′ UTR, thereby impairing RNA synthesis and increasing interferon sensitivity (8). When administered via peripheral immunization, this attenuated ZIKV (ZIKV-LAV) shows satisfying safety and elicits robust immune responses in mice (8). However, when delivered via intracerebral injection, a route commonly used for glioma treatment in clinical settings (38–40), ZIKV-LAV still retains the capacity to infect normal brain tissues in immunodeficient mice (Fig. 6), although the risk is very limited in immunocompetent mice, as shown in our previous studies (16). In comparison, the virus was nearly undetectable in normal brain tissues of mice treated with ZIKV-miR124T (Fig. 6), indicating an increased level of neuroattenuation. Importantly, this safety feature extended beyond intracerebral applications to its performance as a live-attenuated vaccine. In pregnant mouse models, even under conditions where maternal immunity was suppressed by administration of anti-Ifnar1 blocking antibodies, ZIKV-miR124T was associated with significantly lower viral RNA levels in placental and fetal tissues (Fig. 3). These findings clearly demonstrate that ZIKV-miR124T possesses a markedly improved safety profile, thereby expanding the potential for its future clinical applications.

More importantly, ZIKV-miR124T maintains both immunogenicity and oncolytic potency, achieving an ideal balance between safety and efficacy. Attenuation of live viruses requires a delicate balance: enhancing safety while maintaining sufficient replication competence to elicit robust immune or oncolytic responses. Excessive attenuation may compromise efficacy, as illustrated by previous studies of 3′ UTR-deleted chimeric dengue vaccine candidates, which exhibited overly weak replication and failed in animal models or Phase I trials (41, 42). In our study, the replication of ZIKV-miR124T in glioma did not show obvious decrease in glioma cells compared with the WT virus (Fig. 5) and showed only limited decrease in peripheral tissues (Fig. 2), likely attributable to the strategic selection of miR-124, which is almost absent in these tissues (Fig. 1). Therefore, in theory, ZIKV-miR124T is capable of faithfully mimicking wild-type infection during both vaccination and oncolytic treatment, thereby achieving the maximal potential of its immunogenic and oncolytic activities. This may well explain why ZIKV-miR124T exhibited superior oncolytic efficacy compared with ZIKV-LAV and robust immunogenicity, as a single immunization conferred effective protection to both pregnant dams and their offspring.

Beyond its dual functional advantages, ZIKV-miR124T also exhibits excellent translational potential for clinical development. Genetic stability is a critical prerequisite for live-attenuated vaccine and oncolytic platforms. Previous studies have demonstrated that some engineered insertions, deletions, and mutations within the genomes of attenuated viruses are not stable during *in vitro* passaging, illustrating that the stability of live-attenuated virus candidates needs to be carefully monitored and confirmed (43–47). Our data show that ZIKV-miR124T retained the complete integrity of the inserted miR-124T sequence after at least 20 serial passages in both BHK-21 and Vero cells, without any deletions or mutations. The observed stability of ZIKV-miR124T likely results from the rational design of its insertion site, immediately downstream of the ORF stop codon and in the proximal 3′ UTR, which was carefully chosen to preserve the overall structural integrity of the ORF and UTR regions of the viral genome. In addition to its genetic stability, ZIKV-miR124T also demonstrated high replication competence in standard production cell lines, achieving viral titers comparable to those of WT ZIKV in both Vero and BHK-21 cells. In contrast to some attenuated flavivirus strains that exhibit reduced growth efficiency or require specialized culture conditions (8, 48), ZIKV-miR124T maintained strong replication capacity in standard cell lines, an attribute that greatly facilitates scalable vaccine or oncolytic virus production and clinical translation. Together, these properties highlight ZIKV-miR124T as a well-balanced candidate combining safety, stability, and production feasibility—key attributes that support its potential for large-scale development as both a live-attenuated vaccine and an oncolytic platform.

In summary, our study establishes ZIKV-miR124T as a rationally designed, neuron-detargeted ZIKV platform that achieves precise tissue-specific attenuation through miRNA-guided replication control. This strategy addresses the inherent trade-off between safety and efficacy that has limited previous ZIKV vaccine and oncolytic virus candidates. The miR-124T approach represents a paradigm shift in viral attenuation, offering a generalizable framework for developing other tissue-restricted flavivirus platforms. Collectively, our findings highlight ZIKV-miR124T as a next-generation ZIKV derivative with dual potential as a safe live-attenuated vaccine and an effective oncolytic agent against glioma.

## MATERIALS AND METHODS

### Cell lines

Baby hamster kidney fibroblast cell line BHK-21 (ATCC CCL-10), African green monkey kidney cell line Vero (ATCC CCL-81), and Human glioblastoma cell line U251 (NCACC SCSP-559) were cultured in Dulbecco’s modified Eagle’s medium (DMEM, Gibco) containing 10% fetal bovine serum (FBS, Gibco). Aedes albopictus cell line C6/36 (ATCC CRL-1660) was cultured in RPMI 1640 (Gibco) containing 10% FBS. GSCs (387GSC, 456GSC, and 19GSC) were originally isolated and characterized from human GBM surgical specimens as previously described (49–52) and cultured as neurospheres in neurobasal complete media (Gibco) supplemented with 1× B27 (Gibco) (without vitamin A), 2 mM L-glutamine (MacGene), 1 mM sodium pyruvate (MacGene), 10 ng/mL basic fibroblast growth factor (R&D Systems), and 10 ng/mL epidermal growth factor (R&D Systems). DGCs were differentiated from GSCs by withdrawing epidermal growth factor and fibroblast growth factor and adding 10% fetal bovine serum (FBS; MacGene). DGCs were cultured in Dulbecco’s modified Eagle’s medium (MacGene) containing 10% FBS. Neural progenitor cells (NPCs) and neurons were induced from the embryonic stem cell line (NSCRC CB0003) and provided by the Organcare (Shenzhen) Biotechnology Company, Shenzhen, China. Mammalian cells (including NPCs and neurons) were grown at 37°C with 5% CO2, whereas C6/36 cells were maintained at 28°C.

### Small RNA sequencing and differential expression analysis

Total RNA in each sample was extracted using TRIzol reagent. RNA concentration and integrity were assessed using the Agilent 2100 Bioanalyzer (Agilent Technologies, USA). For small RNA sequencing, miRNA libraries were prepared using the VAHTS Small RNA Library Prep Kit for Illumina V2 (Vazyme, China). Library quality was evaluated using the Agilent 2100 system, and sequencing was performed on an Illumina platform (single-end 1 × 50 bp) at Novogene (Beijing, China). Raw sequencing data in FASTQ format was first processed using customized Perl and Python scripts. In this step, reads containing poly-N, 5′ adaptor contaminants, missing 3′ adaptors or insert tags, reads with long homopolymeric stretches (poly-A/T/G/C), and other low-quality reads were removed to generate the final set of clean reads. Clean reads were mapped to the human reference genome (hg38) and to known mature miRNAs from miRBase release 22 using Bowtie. The number of reads mapped to each miRNA was quantified to generate expression count matrices. Differential expression analysis of miRNAs among different cell types was performed using the DESeq2 package (v1.42.0) in R.

### ZIKV strains and mutant construction

The WT ZIKV strain FSS13025 (GenBank KU955593.1) was originally isolated from a patient in Cambodia in 2010 and recovered from an infectious cDNA clone (pFLZIKV) (53). This strain served as the backbone for the introduction of the miRNA target sequences immediately after the TAA stop codon into the ZIKV genome, between nucleotides 10379 and 10380, using overlapping PCR methods. All the mutants were confirmed by restriction endonuclease digestion and DNA sequencing. The infectious clone plasmids that had been sequence-verified were linearized with Cla I (New England Biolabs) and purified by phenol/chloroform extraction. *In vitro*-transcribed viral RNA was prepared using a Ribomax T7 large-scale RNA production kit (Promega) and purified using a Purelink RNA Mini Kit (Thermo Fisher Scientific). The RNA was then transfected into BHK-21 cells using Lipofectamine 3000 reagent (Thermo Fisher Scientific), according to the manufacturer’s instructions, and the culture supernatants were collected at 48–72 h post-transfection. ZIKV stocks were prepared in C6/36 cells, titers were measured by a standard plaque-forming assay on BHK-21 cells, and mutations were confirmed by RT-PCR and DNA sequencing. All viral stocks were stored in aliquots at −80°C until use.

### Indirect immunofluorescence assay (IFA)

BHK-21 cells seeded in 24-well plates were transfected with viral RNA. At 48–72 h post-transfection, the infected cells were directly used for IFA. The cells were fixed in acetone/methanol (vol/vol, 3:7) at −20°C. The fixed cells were incubated with the mouse anti-ZIKV E protein mAb (1:1,500 dilution, Cat. no. BF-1176-56, BioFront Technologies) at 37°C for 1 h and later washed three times with PBS. They were then incubated with secondary antibodies conjugated to Alexa Fluor 488 (anti-mouse IgG, 1:200 dilutions, Zsbio) in PBS for 1 h at 37°C and washed again as described above. For cell nuclei staining, DAPI (0.5 ng/μL) was added to the cells and incubated for 10 min at RT.

### Growth curves

BHK-21 and C6/36 were directly seeded into 24-well plates, while NPCs and GSCs were seeded into 24-well plates pretreated with Matrigel (BD). After 24 h, BHK-21 and C6/36 were infected with viruses at a multiplicity of infection (MOI) of 0.01, and the NPCs and GSCs were infected at an MOI of 1. One hour later, the culture medium was refreshed, and the culture supernatants were collected at the indicated time points after infection. Viral RNA in the culture supernatants was extracted using PureLink RNA Mini Kit (Thermo Fisher Scientific) and quantified by RT-qPCR with primers and probes targeting the E gene of ZIKV: F-(5′-CCGCTGCCCAACACAAG-3′); R-(5′- CCACTAACGTTCTTTTGCAGACAT-3′); P-(5′- AGCCTACCTTGACAAGCAGTCAGACACTCAA-3′). Infectious virus titers were determined by standard plaque assay on BHK-21 cells. Briefly, culture supernatants were subjected to serial 10-fold dilutions in DMEM supplemented with 2% FBS. Four hundred microliters of each dilution was inoculated onto BHK-21 cell monolayers in 12-well plates and incubated at 37°C with 5% CO₂ for 2 h. After incubation, the inoculum was removed, and the cells were gently washed three times with PBS. The cells were overlaid with 1 mL of DMEM containing 1% low-melting-point agarose and 2% FBS. Plates were incubated for 4 days, after which the cells were fixed with 4% formaldehyde at room temperature and stained with 1% crystal violet. Plaques were enumerated to calculate viral titers, expressed as plaque-forming units per milliliter (PFU/mL).

### Genetic stability assay

The recovered viruses were consecutively passaged in BHK-21 and Vero cells for 20 passages. The viral RNA of passaged viruses was extracted by using the PureLink RNA Mini Kit (Thermo Fisher Scientific), and the mutations were confirmed by RT-PCR and DNA sequencing.

### miRNA mimic transfection

The miR-124 mimic and non-specific control mimic were purchased from Ambion (Thermo Fisher Scientific). Vero cells were seeded into 24-well plates and transfected with miR-124 mimic or negative control at 20 pmol/well using Lipofectamine 3000 (Thermo Fisher Scientific), according to the manufacturer’s protocol. At 6 h post-transfection, cells were inoculated with WT, ZIKV-miR124T, or ZIKV-miR124C at an MOI of 0.01. At the indicated time points after infection, culture supernatants were collected, and viral RNA amounts were determined by RT-qPCR.

### Enzyme-linked immunosorbent assay (ELISA)

ELISA was used to measure the IgG antibody titers in mouse sera. Briefly, 96-well plates were coated with recombinant ZIKV E protein (54) at 50 ng/well in coating buffer overnight at 4°C. The coated plates were blocked with 5% skim milk, then incubated with serial dilutions of serum at 37°C for 1 h and later washed three times with PBST. Subsequently, the plates were incubated with secondary antibodies conjugated to HRP (anti-mouse IgG, 1: 20,000 dilutions, Zsbio) for 1 h at 37°C. Then, the plates were washed again as described above and followed by incubation with TMB substrate (CWBIO). The absorbance at 450 nm was measured, and 2.1 times the negative control was considered positive.

### Neutralization assay

Neutralizing antibody titers were determined by a standard plaque reduction neutralization test (PRNT). In brief, BHK-21 cells were seeded in 12-well plates and cultured for approximately 16 h until 90–100% confluence. Serial 3-fold dilutions of serum were prepared in a 48-well plate and pre-incubated with WT virus in a 1:1 (vol/vol) ratio to generate a mixture containing ~100 PFU/mL of viruses for 1 h at 37°C, and DMEM was used as a negative control. The virus/serum mixtures were added to the Vero cells and incubated for 1 h at 37°C. Then, the mixtures were removed and replaced with DMEM containing 2% FBS and 1% low-melting-point agarose (Promega). Four days later, the cells were fixed with 4% formaldehyde and stained with 0.2% crystal violet. The 50% neutralization titer (PRNT_50_) was calculated by the method of Spearman-Karber.

### Mouse experiments

Mice were maintained at a constant ambient temperature in a 12-hour day/night cycle and fed *ad libitum* in a specific-pathogen-free facility. A129 (129/SvEv mice deficient in IFN-α/β receptor) mice used in this study were raised in the Animal Laboratory Animal Center, AMMS. BALB/c mice and BALB/c nude mice were purchased from Beijing Vital River Laboratory Animal Technology Co., Ltd.

### Adult A129 mouse model

For the neuroinvasiveness test, groups of 4-week-old A129 mice were injected with 10^4^ PFU of WT or ZIKV-miR124T via the intraperitoneal route. Body weight was recorded for 14 days. Blood was collected at different time points post-infection for determination of viral RNA loads by RT-qPCR. Mouse brain, testis, liver, and rectum were collected on day 7 post-infection and subjected to RT-qPCR. For evaluation of immunogenicity and immune protection, groups of 4-week-old A129 mice were immunized with 10^4^ PFU of ZIKV-miR124T virus or PBS via the subcutaneous route. On day 24 after immunization, mouse sera were quantified for IgG titer and PRNT_50_. On day 26 after immunization, mice were challenged via the intraperitoneal route with 10^5^ PFU of WT virus. The challenged mice were monitored for viremia for 7 days, weight loss for 14 days, and survival for 30 days after challenge.

### Pregnant BALB/c mouse model

For vertical transmission test, groups of 9-week-old BALB/c female mice were administered 2.0 mg of anti-Ifnar1-blocking antibody at E5.5 and infected with WT or ZIKV-miR124T at E6.5. Maternal sera, brains, placenta, and fetal heads were collected at E13.5, and viral RNA amounts were determined by RT-qPCR. For evaluation of immunogenicity and immune protection, groups of 6-week-old BALB/c female mice were immunized with 10^6^ PFU of ZIKV-miR124T virus or PBS via the subcutaneous route. On day 24 after immunization, mouse sera were quantified for PRNT_50_. On day 26 after immunization, the immunized female mice were mated with BALB/c males. Pregnant mice were injected with 2.0 mg of anti-Ifnar1-blocking antibody at E5.5 and challenged with 10^5^ PFU of WT virus at E6.5. Mouse sera were collected from E7.5 to E9.5, and maternal brains and spleens, placenta, and fetal heads were collected at E13.5 and E18.5, respectively. On day 8 after birth, the suckling mice were intracerebrally injected with 10^5^ PFU of WT virus, while another group of 8-day-old suckling mice received PBS as a mock control. The brains were collected on day 22 after infection. Viral RNA amounts in tissues were determined by RT-qPCR.

### Neonatal mouse model

For neurovirulence test, groups of 1-day-old neonatal BALB/c mice were inoculated with 5 × 10^4^ PFU of WT or mutant viruses via the intracerebral route, and the survival curves were monitored and recorded for 30 days. For the test of viral replication in mouse brain, 5 × 10^3^ PFU of WT or ZIKV-miR124T virus were inoculated intracerebrally into 1-day-old neonatal BALB/c mice. On days 3, 6, 9, and 12 after infection, brains were collected, and viral RNA amounts were determined by RT-qPCR.

### Construction of heterotopic xenograft mouse model

A total of 10^6^ GSCs were subcutaneously implanted into the flanks of 3-week-old BALB/c nude mice. On day 6 post-transplantation, mice were intratumorally injected with 1 × 10^5^ PFU of ZIKV-miR124T or PBS. The volume of tumors was measured at the indicated time points after intratumoral virus administration. Subcutaneous tumors were surgically excised and measured 6 days after intratumoral virus administration, and viral RNA amounts in the samples were determined by RT-qPCR.

### Construction of orthotopic xenograft mouse model

A total of 5 × 10^4^ luciferase-labeled GSCs were co-implanted with 10^4^ PFU of ZIKV-miR124T, ZIKV-LAV, or RPMI 1640 intracerebrally into the right frontal lobe of a nude mouse. After implantation, bioluminescence imaging was performed using a charge-coupled device camera (Xenogen Corp). Briefly, 1.5 mg of the substrate D-LUCIFERIN sodium salt (Gold Biotechnology) was administered intraperitoneally to each mouse, and images were acquired 9 min later for 60 s. To quantify the amount of light emitted from the tumor, regions of interest (ROIs) were manually defined after imaging, and the photon flux was calculated (in photons/second/ square centimeter/steradian) using Living Image 3.0 (Caliper Life Sciences). For experiments assessing survival, mice were monitored until the last mouse showed neurological signs.

### Hematoxylin and eosin (H&E) and immunofluorescence staining of brain tissues

At indicated time points, mouse brains were harvested and prepared in 5 µm thick cryosections. The cryosections were stained with hematoxylin and eosin and subjected to histological examination. Brain cryosections were incubated with a primary mouse anti-ZIKV E protein antibody (Abcam) (1:500 dilution) overnight at 4°C. The sections were then incubated with Alexa Fluor 488-conjugated anti-mouse secondary antibodies (Thermo Fisher) (1:300 dilution) for 2 h at 37°C. Finally, the sections were incubated with DAPI (4’,6-diamidino-2-phenylindole) (1:1,000 dilution) for 10 min. Images were acquired using confocal laser scanning microscopy (Zeiss).

### Statistical analysis

All data were analyzed using GraphPad Prism software. Results were expressed as the mean ± SD or as described in the corresponding legends. Log-rank tests were performed for the survival analysis. For other results, statistical analysis was performed by Student’s unpaired *t*-test.

## Data Availability

Small RNA sequencing data have been deposited in the NCBI GEO database under accession no. GSE331013. All relevant data from this study are available from the authors upon request.

## SUPPLEMENTAL MATERIAL

Supplemental figures jvi.00208-26-s0001.pdf Fig. S1 to S3.

ASM does not own the copyrights to Supplemental Material that may be linked to, or accessed through, an article. The authors have granted ASM a non-exclusive, world-wide license to publish the Supplemental Material files. Please contact the corresponding author directly for reuse.
