# Supplementary material for: MicroRNA-124-targeted recombinant Zika virus: a dual-functional and safe candidate for vaccination and oncolytic virotherapy
Source: J Virol. 2026 May 15;100(6):e00208-26. doi: 10.1128/jvi.00208-26 (PMC13288617; doi:10.1128/jvi.00208-26)
Supplement: Supplemental figures — Fig. S1 to S3. [file jvi.00208-26-s0001.pdf]

Fig S1

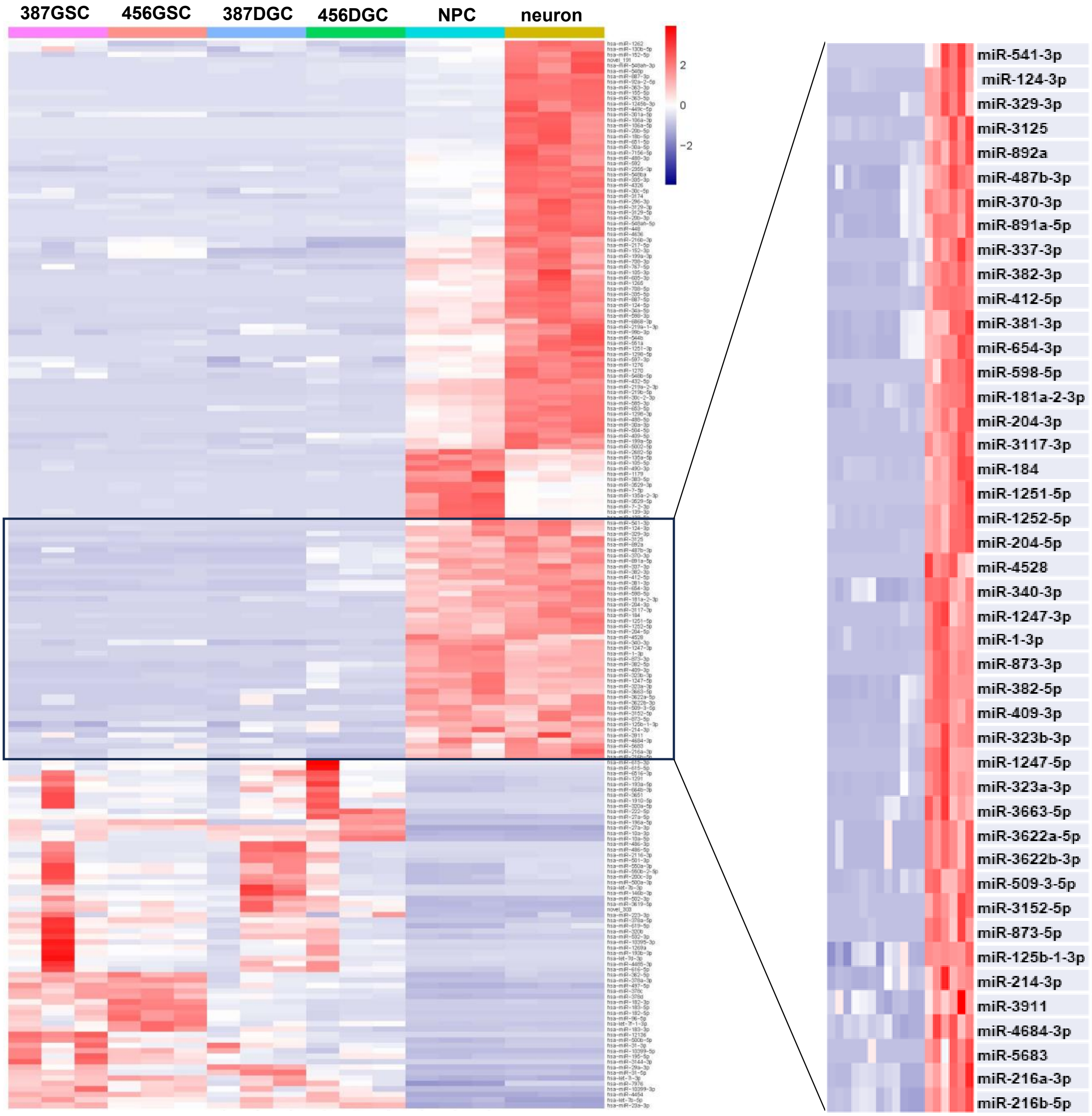

FIG S1 Differential miRNA expression between human neural cells and glioma cells. Small RNA sequencing was performed to determine miRNA expression profiles in glioma stem-like cells (GSCs), differentiated GSCs (DGCs), neural progenitor cells (NPCs), and neurons. The heatmap shows differential miRNA expression between glioma cells (GSCs and DGCs) and neural cells (NPCs and neurons). Data are row-standardized (Z-score); blue indicates low expression, white indicates mean expression, and red indicates high expression. The boxed region highlights miRNAs highly enriched in NPCs and neurons but minimally expressed in GSCs and DGCs.

Fig S2

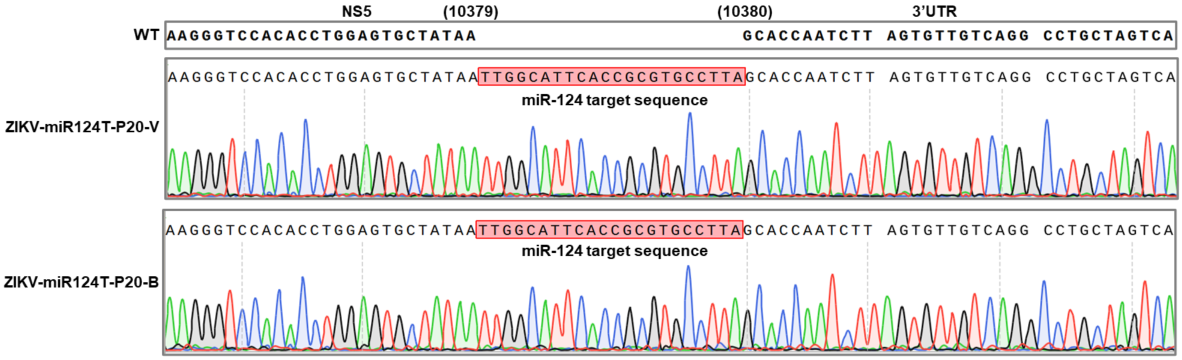

FIG S2 Genetic stability of the miR-124 target insertion during serial passaging. Representative Sanger sequencing chromatograms spanning the region containing the miR-124 target sequence (miR124T) are shown for ZIKV-miR124T after 20 serial passages in Vero (ZIKV-miR124T-P20-V) and BHK-21 (ZIKV-miR124T-P20-B). The miR-124 target sequence is highlighted in red.

Fig S3

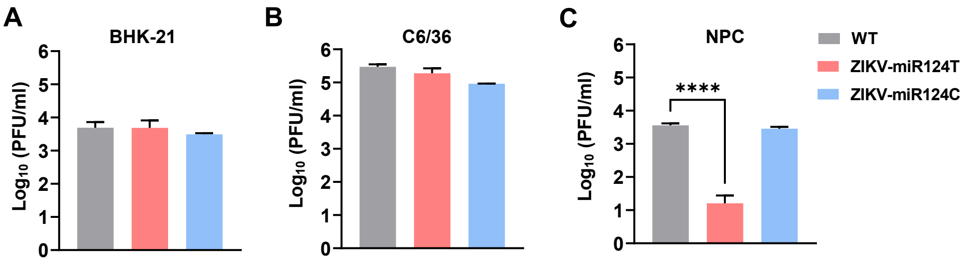

FIG S3 Comparison of infectious viral titers of WT, ZIKV-miR124T and ZIKV-miR124C in supernatants from infected BHK-21, C6/36 or NPCs. Viral titers in culture supernatants collected from BHK-21 (A), C6/36 (B), and NPCs (C) infected with WT, ZIKV-miR124T or ZIKV-miR124C at 72 h post-infection were determined by plaque assay on BHK-21 cells. These data correspond to the final time point (72 h post-infection) of the viral RNA growth kinetics shown in Fig 1. Data are presented as mean ± SD. Student's t-test was used for statistical analysis. ****, $p<0.0001$.
